# Melaminium sulfate

**DOI:** 10.1107/S1600536807067463

**Published:** 2008-01-04

**Authors:** Bao-Yong Zhu, De-Liang Cui, Hai-Peng Jing

**Affiliations:** a Institute of Crystalline Materials, Shandong University, Jinan 250100, People’s Republic of China

## Abstract

In the title compound, C_3_H_8_N_6_^2+^·SO_4_^2−^, the melaminium cations and sulfate anions are inter­connected by N—H⋯N and N—H⋯O hydrogen bonds, forming a layer in the (101) plane. The layers are connected through multiple hydrogen bonds and π–π stacking inter­actions (centroid–centroid distance of about 3.4 Å).

## Related literature

For related literature, see: Janczak & Perpétuo (2001*a* ▶,*b* ▶); Martin & Pinkerton (1995 ▶); Dewar *et al.* (1985 ▶).
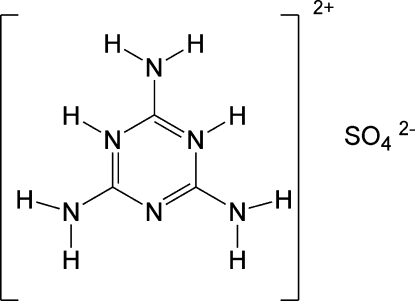


## Experimental

### 

#### Crystal data

C_3_H_8_N_6_^2+^·SO_4_^2−^
*M* *_r_* = 224.21
Monoclinic, 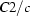

*a* = 18.5787 (3) Å
*b* = 8.6272 (2) Å
*c* = 12.7945 (4) Å
β = 129.739 (1)°
*V* = 1576.94 (7) Å^3^
*Z* = 8
Mo *K*α radiation
μ = 0.42 mm^−1^
*T* = 293 (2) K
0.32 × 0.27 × 0.26 mm

#### Data collection

Bruker APEXII CCD diffractometer
Absorption correction: multi-scan (*APEX2*; Bruker, 2005 ▶) *T*_min_ = 0.878, *T*_max_ = 0.900
3793 measured reflections
1794 independent reflections
1672 reflections with *I* > 2σ(*I*)
*R*_int_ = 0.013

#### Refinement

*R*[*F* ^2^ > 2σ(*F* ^2^)] = 0.031
*wR*(*F* ^2^) = 0.088
*S* = 1.00
1794 reflections
136 parameters
H atoms treated by a mixture of independent and constrained refinement
Δρ_max_ = 0.48 e Å^−3^
Δρ_min_ = −0.45 e Å^−3^

### 

Data collection: *APEX2* (Bruker, 2005 ▶); cell refinement: *APEX2*; data reduction: *APEX2*; program(s) used to solve structure: *SIR97* (Altomare *et al.*, 1999 ▶); program(s) used to refine structure: *SHELXL97* (Sheldrick, 1997 ▶); molecular graphics: *SHELXTL* (Bruker, 1997 ▶); software used to prepare material for publication: *WinGX* (Farrugia, 1999 ▶).

## Supplementary Material

Crystal structure: contains datablocks global, I. DOI: 10.1107/S1600536807067463/bt2656sup1.cif

Structure factors: contains datablocks I. DOI: 10.1107/S1600536807067463/bt2656Isup2.hkl

Additional supplementary materials:  crystallographic information; 3D view; checkCIF report

## Figures and Tables

**Table 1:** Hydrogen-bond geometry (Å, °)

*D*—H⋯*A*	*D*—H	H⋯*A*	*D*⋯*A*	*D*—H⋯*A*
N6—H6⋯O2^i^	0.87 (3)	1.76 (3)	2.622 (2)	171 (3)
N5—H5⋯O4^ii^	0.85 (3)	1.76 (3)	2.608 (2)	176 (3)
N3—H3*B*⋯O2^i^	0.86	2.59	3.244 (2)	134
N3—H3*B*⋯O1^iii^	0.86	2.44	2.944 (2)	118
N3—H3*A*⋯N4^iv^	0.86	2.14	3.000 (2)	176
N2—H2*B*⋯O3^i^	0.86	1.97	2.822 (2)	172
N2—H2*A*⋯O3^v^	0.86	2.02	2.836 (2)	159
N1—H1*B*⋯O2^ii^	0.86	1.99	2.838 (2)	169
N1—H1*A*⋯O1^vi^	0.86	2.43	2.992 (2)	123
N1—H1*A*⋯O1^vii^	0.86	2.11	2.887 (2)	151

Symmetry codes: (i) 
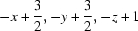
; (ii) 
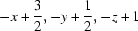
; (iii) 
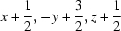
; (iv) 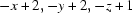; (v) 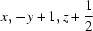; (vi) 
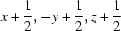
; (vii) 
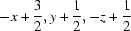
.

**Fig. 1.**
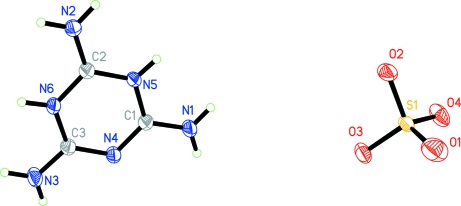
View of the asymmetric unit of (I) showing the atom-labelling scheme. Displacement ellipsoids are drawn at the 50% probability level. H atoms are represented by circles of arbitrary size.

**Fig. 2.**
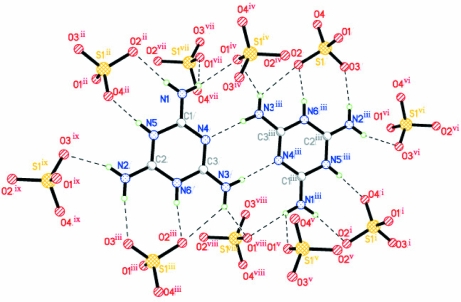
Diagram showing the hydrogen bonds of the melaminium cation. symmetry codes: (i) x, y + 1, z; (ii) -x + 1, -y, -z + 1; (iii) -x + 1, -y + 1, -z + 1; (iv) -x + 1, y, -z + 1/2; (v) -x + 1, y + 1, -z + 1/2; (vi) -x + 3/2, y + 1/2, -z + 1/2; (vii) x, -y, z + 1/2; (viii) x, -y + 1, z + 1/2; (ix) x - 1/2, -y + 1/2, z + 1/2.

**Fig. 3.**
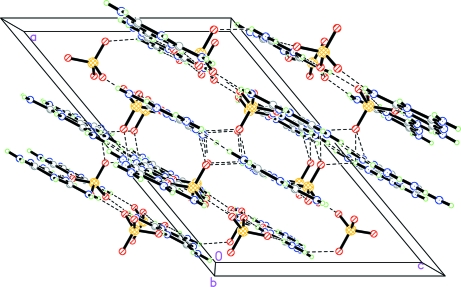
The molecular packing of (I) in the unit cell showing the hydrogen-bonding interaction (dashed lines).

## supplementary crystallographic information

### Comment

Salts of melamine and its derivatives can develop supramolecular structures *via* hydrogen bonding by self assembly. Monoprotonated melaminium sulfate hydrate, (C_3_H_7_N_6_)_2_ SO_4_.H_2_O, has been structurally investigated (Janczak & Perpétuo, 2001*b*). We present here the solid state structure of anhydrous diprotonated melaminium salt.

The internal C—N—C angle at the protonated N atoms [C2—N6—C3 120.35 (15) ° and C2—N5—C1 120.25 (15) °] is significantly larger than the C—N—C angle at the non-protonated N atom [C1—N4—C3 116.79 (15) °]. These differences are due to the steric effect of the lone-pair electrons and are fully consistent with the valence-shell electron-pair repulsion theory (Janczak & Perpétuo, 2001*a*). The two shortest bonds [N4—C3 1.333 (2) Å and N4—C1 1.338 (2) Å] are those furthest from the protonated ring N atoms. The two longest bonds [N6—C3 1.367 (2) Å and N5—C1 1.373 (2) Å] are those connected to the shortest bonds. This has the effect of opening up the ring bond angles at atoms C1 and C3, thus creating the largest bond angles in the ring [N4—C3—N6 122.4 (1) ° and N4—C1—N5 122.1 (1) °]. A semi-empirical calculation, with the AM1 parameter set (Dewar *et al.*, 1985) on the melaminium residue diprotonated at two ring N atoms, results in almost the same geometrical features as being found in the title compound. The distortion of the aromatic ring is quite similar to that reported for the melaminium diperchlorate monohydrate salt (Martin & Pinkerton, 1995), as well as for melaminium bis(4-hydroxybenzene-sulfonate) dihydrate (Janczak & Perpétuo, 2001*a*).

The melaminium residue is involved in eleven hydrogen bonds, two N—H···N bonds with the neighbouring melaminium residue and nine N—H···O bonds with six neighbouring SO_4_^2-^ anions. Two of the SO_4_^2-^ anions are acceptors of two and three hydrogen bonds, respectively, while the other four are acceptors of one hydrogen bond each (Fig. 2). The H atoms at the protonated N atoms of the melaminium residue are involved in almost linear N—H···O hydrogen bonds.

Each SO_4_^2-^ ion is involved as an acceptor in nine hydrogen bonds connecting to six melaminium residues. The O1 and O2 atoms are the most interesting ones as they all accept three hydrogen atoms each. O3 forms two hydrogen bonds with melaminium residues *via* the H atoms of the amino groups, and O4 forms only one hydrogen bond *via* H5 atom at the protonated N atom of the melaminium residue.

The melaminium residues are interconnected by two almost linear N—H···N hydrogen bonds and five N—H···O hydrogen bonds. The distance between the centroids of the aromatic rings in adjacent layers (symmetry operator 2 - *x*, +*y*, 1.5 - *z*) is about 3.4 Å, which is much shorter than the maximum distance for the π-π stacking interaction (3.8 Å for centroid-centroid distance), indicating strong π-π stacking interactions. The two-dimensional layers are extensively interconnected by multiple hydrongen bonds with sulfate anions and π-π stacking interactions (Fig. 3).

### Experimental

0.126 g (0.001 mol) of melamine was dissolved in 50 ml hot water. To this solution 4 ml 98% sulfate acid was slowly added. After several days, colorless crystals of (I) appeared.

### Refinement

The H atoms bonded to the ring N atoms were located in difference Fourier map and their positions and displacement parameters were refined freely. The amino H atoms were added geometrically and treated as riding, with N—H = 0.86 Å and *U*_iso_(H) = 1.2*U*_eq_(N).

### Crystal data

**Table:**

C_3_H_8_N_6_^2+^·SO_4_^2–^	*F*_000_ = 928
*M**_r_* = 224.21	*D*_x_ = 1.889 Mg m^−^^3^
Monoclinic, *C*2/*c*	Mo *K*α radiation λ = 0.71073 Å
*a* = 18.5787 (3) Å	Cell parameters from 2920 reflections
*b* = 8.6272 (2) Å	θ = 2.8–27.5º
*c* = 12.7945 (4) Å	µ = 0.42 mm^−^^1^
β = 129.7390 (10)º	*T* = 293 (2) K
*V* = 1576.94 (7) Å^3^	Prism, colorless
*Z* = 8	0.32 × 0.27 × 0.26 mm

### Data collection

**Table:**

Bruker APEX2 CCD diffractometer	1794 independent reflections
Radiation source: fine-focus sealed tube	1672 reflections with *I* > 2σ(*I*)
Monochromator: graphite	*R*_int_ = 0.013
*T* = 293(2) K	θ_max_ = 27.5º
φ and ω scans	θ_min_ = 2.8º
Absorption correction: multi-scan(APEX2; Bruker, 2005)	*h* = −24→19
*T*_min_ = 0.879, *T*_max_ = 0.900	*k* = −6→11
3793 measured reflections	*l* = −16→16

### Refinement

**Table:**

Refinement on *F*^2^	Hydrogen site location: inferred from neighbouring sites
Least-squares matrix: full	H atoms treated by a mixture of independent and constrained refinement
*R*[*F*^2^ > 2σ(*F*^2^)] = 0.031	*w* = 1/[σ^2^(*F*_o_^2^) + (0.041*P*)^2^ + 3.6165*P*] where *P* = (*F*_o_^2^ + 2*F*_c_^2^)/3
*wR*(*F*^2^) = 0.089	(Δ/σ)_max_ < 0.001
*S* = 1.00	Δρ_max_ = 0.48 e Å^−^^3^
1794 reflections	Δρ_min_ = −0.45 e Å^−^^3^
136 parameters	Extinction correction: SHELXL97 (Sheldrick, 1997), Fc^*^=kFc[1+0.001xFc^2^λ^3^/sin(2θ)]^-1/4^
Primary atom site location: structure-invariant direct methods	Extinction coefficient: 0.0037 (5)
Secondary atom site location: difference Fourier map	

### Special details

**Table:**

Geometry. All e.s.d.'s (except the e.s.d. in the dihedral angle between two l.s. planes) are estimated using the full covariance matrix. The cell e.s.d.'s are taken into account individually in the estimation of e.s.d.'s in distances, angles and torsion angles; correlations between e.s.d.'s in cell parameters are only used when they are defined by crystal symmetry. An approximate (isotropic) treatment of cell e.s.d.'s is used for estimating e.s.d.'s involving l.s. planes.
Refinement. Refinement of *F*^2^ against ALL reflections. The weighted *R*-factor *wR* and goodness of fit *S* are based on *F*^2^, conventional *R*-factors *R* are based on *F*, with *F* set to zero for negative *F*^2^. The threshold expression of *F*^2^ > σ(*F*^2^) is used only for calculating *R*-factors(gt) *etc*. and is not relevant to the choice of reflections for refinement. *R*-factors based on *F*^2^ are statistically about twice as large as those based on *F*, and *R*- factors based on ALL data will be even larger.

### Fractional atomic coordinates and isotropic or equivalent isotropic displacement parameters (Å^2^)

**Table:**

	*x*	*y*	*z*	*U*_iso_*/*U*_eq_	
S1	0.65733 (3)	0.06360 (5)	0.27385 (4)	0.02105 (15)	
O2	0.63929 (12)	0.11467 (16)	0.36667 (16)	0.0369 (4)	
O4	0.70593 (10)	−0.08710 (15)	0.32413 (17)	0.0348 (4)	
C2	0.84604 (12)	0.9717 (2)	0.65207 (17)	0.0210 (3)	
N5	0.86032 (10)	0.82418 (17)	0.63479 (15)	0.0215 (3)	
N3	0.95352 (12)	1.16685 (18)	0.54108 (18)	0.0312 (4)	
H3A	0.9834	1.1488	0.5118	0.037*	
H3B	0.9448	1.2607	0.5534	0.037*	
O3	0.71951 (10)	0.17704 (15)	0.28119 (15)	0.0309 (3)	
N2	0.80592 (11)	1.00382 (18)	0.70342 (17)	0.0277 (3)	
H2A	0.7879	0.9301	0.7272	0.033*	
H2B	0.7974	1.0988	0.7136	0.033*	
C3	0.92125 (12)	1.0513 (2)	0.56700 (18)	0.0222 (3)	
N4	0.93490 (11)	0.90603 (17)	0.54780 (16)	0.0236 (3)	
N1	0.91024 (13)	0.64908 (18)	0.55885 (18)	0.0317 (4)	
H1A	0.9372	0.6259	0.5259	0.038*	
H1B	0.8889	0.5768	0.5787	0.038*	
O1	0.56934 (11)	0.0514 (2)	0.13681 (16)	0.0440 (4)	
N6	0.87638 (11)	1.08607 (18)	0.61675 (16)	0.0232 (3)	
C1	0.90174 (12)	0.7934 (2)	0.57868 (17)	0.0219 (3)	
H6	0.8684 (18)	1.183 (3)	0.626 (2)	0.042 (7)*	
H5	0.8388 (18)	0.749 (3)	0.651 (3)	0.044 (7)*	

### Atomic displacement parameters (Å^2^)

**Table:**

	*U*^11^	*U*^22^	*U*^33^	*U*^12^	*U*^13^	*U*^23^
S1	0.0277 (2)	0.0160 (2)	0.0288 (2)	0.00029 (15)	0.0224 (2)	0.00143 (15)
O2	0.0657 (10)	0.0212 (7)	0.0574 (9)	−0.0009 (7)	0.0549 (9)	−0.0015 (6)
O4	0.0425 (8)	0.0182 (6)	0.0605 (10)	0.0067 (6)	0.0408 (8)	0.0085 (6)
C2	0.0228 (8)	0.0197 (8)	0.0233 (8)	−0.0007 (6)	0.0160 (7)	−0.0004 (6)
N5	0.0284 (7)	0.0166 (7)	0.0289 (7)	−0.0010 (6)	0.0226 (7)	0.0004 (6)
N3	0.0438 (9)	0.0183 (7)	0.0528 (10)	−0.0030 (7)	0.0407 (9)	−0.0011 (7)
O3	0.0416 (8)	0.0189 (6)	0.0502 (8)	−0.0047 (5)	0.0377 (7)	−0.0022 (6)
N2	0.0395 (9)	0.0202 (7)	0.0418 (9)	−0.0002 (6)	0.0345 (8)	−0.0006 (6)
C3	0.0246 (8)	0.0201 (8)	0.0274 (8)	−0.0013 (6)	0.0191 (7)	−0.0010 (6)
N4	0.0296 (8)	0.0185 (7)	0.0345 (8)	−0.0010 (6)	0.0259 (7)	−0.0010 (6)
N1	0.0519 (10)	0.0172 (7)	0.0516 (10)	−0.0016 (7)	0.0449 (9)	−0.0018 (7)
O1	0.0361 (8)	0.0554 (10)	0.0329 (8)	−0.0085 (7)	0.0185 (7)	0.0045 (7)
N6	0.0320 (8)	0.0156 (7)	0.0330 (8)	−0.0002 (6)	0.0259 (7)	−0.0012 (6)
C1	0.0254 (8)	0.0194 (8)	0.0262 (8)	−0.0003 (6)	0.0188 (7)	−0.0007 (6)

### Geometric parameters (Å, °)

**Table:**

S1—O1	1.447 (1)	N3—H3B	0.8600
S1—O3	1.471 (1)	N2—H2A	0.8600
S1—O4	1.475 (1)	N2—H2B	0.8600
S1—O2	1.492 (1)	C3—N4	1.333 (2)
C2—N2	1.300 (2)	C3—N6	1.367 (2)
C2—N5	1.347 (2)	N4—C1	1.338 (2)
C2—N6	1.350 (2)	N1—C1	1.300 (2)
N5—C1	1.373 (2)	N1—H1A	0.8600
N5—H5	0.85 (3)	N1—H1B	0.8600
N3—C3	1.310 (2)	N6—H6	0.87 (3)
N3—H3A	0.8600		
			
O1—S1—O3	111.02 (9)	C2—N2—H2B	120.0
O1—S1—O4	111.51 (10)	H2A—N2—H2B	120.0
O3—S1—O4	108.77 (8)	N3—C3—N4	119.80 (16)
O1—S1—O2	109.36 (10)	N3—C3—N6	117.71 (16)
O3—S1—O2	108.65 (8)	N4—C3—N6	122.46 (16)
O4—S1—O2	107.42 (9)	C3—N4—C1	116.79 (15)
N2—C2—N5	121.41 (16)	C1—N1—H1A	120.0
N2—C2—N6	120.69 (16)	C1—N1—H1B	120.0
N5—C2—N6	117.88 (15)	H1A—N1—H1B	120.0
C2—N5—C1	120.25 (15)	C2—N6—C3	120.35 (15)
C2—N5—H5	120.6 (18)	C2—N6—H6	120.8 (17)
C1—N5—H5	118.9 (19)	C3—N6—H6	118.8 (17)
C3—N3—H3A	120.0	N1—C1—N4	120.09 (16)
C3—N3—H3B	120.0	N1—C1—N5	117.76 (16)
H3A—N3—H3B	120.0	N4—C1—N5	122.13 (15)
C2—N2—H2A	120.0		
			
N2—C2—N5—C1	179.14 (16)	N3—C3—N6—C2	−176.50 (17)
N6—C2—N5—C1	−2.1 (2)	N4—C3—N6—C2	1.8 (3)
N3—C3—N4—C1	178.76 (17)	C3—N4—C1—N1	178.06 (18)
N6—C3—N4—C1	0.5 (3)	C3—N4—C1—N5	−3.6 (3)
N2—C2—N6—C3	177.84 (17)	C2—N5—C1—N1	−177.10 (17)
N5—C2—N6—C3	−0.9 (3)	C2—N5—C1—N4	4.6 (3)

### Hydrogen-bond geometry (Å, °)

**Table:**

*D*—H···*A*	*D*—H	H···*A*	*D*···*A*	*D*—H···*A*
N6—H6···O2^i^	0.87 (3)	1.76 (3)	2.622 (2)	171 (3)
N5—H5···O4^ii^	0.85 (3)	1.76 (3)	2.608 (2)	176 (3)
N3—H3B···O2^i^	0.86	2.59	3.244 (2)	134
N3—H3B···O1^iii^	0.86	2.44	2.944 (2)	118
N3—H3A···N4^iv^	0.86	2.14	3.000 (2)	176
N2—H2B···O3^i^	0.86	1.97	2.822 (2)	172
N2—H2A···O3^v^	0.86	2.02	2.836 (2)	159
N1—H1B···O2^ii^	0.86	1.99	2.838 (2)	169
N1—H1A···O1^vi^	0.86	2.43	2.992 (2)	123
N1—H1A···O1^vii^	0.86	2.11	2.887 (2)	151

Symmetry codes: (i) −*x*+3/2, −*y*+3/2, −*z*+1; (ii) −*x*+3/2, −*y*+1/2, −*z*+1; (iii) *x*+1/2, −*y*+3/2, *z*+1/2; (iv) −*x*+2, −*y*+2, −*z*+1; (v) *x*, −*y*+1, *z*+1/2; (vi) *x*+1/2, −*y*+1/2, *z*+1/2; (vii) −*x*+3/2, *y*+1/2, −*z*+1/2.
